# Causalidade e Gravidade das Reações Adversas e Alterações Laboratoriais ao Tratamento com Benznidazol em Pacientes com Doença de Chagas Crônica

**DOI:** 10.36660/abc.20230787

**Published:** 2024-09-06

**Authors:** Alanna Carla da Costa Belmino, Emanuela Kelly Silva Sousa, José Damião da Silva, Eduardo Arrais Rocha, Francisca Mylena Melgaço Nunes, Tiago Lima Sampaio, Leones Fernandes Evangelista, Bruna Ribeiro Duque, Isabel Cristina da Silva Araújo, José Igor de Oliveira Jacó, Maria de Fátima Oliveira

**Affiliations:** 1 Universidade Federal do Ceará Fortaleza CE Brasil Universidade Federal do Ceará, Fortaleza, CE – Brasil

**Keywords:** Doença de Chagas, Efeitos Colaterais e Reações Adversas Relacionados a Medicamentos, Exames e Diagnósticos Laboratoriais

## Abstract

**Fundamento:**

A Doença de Chagas (DC) representa um grave problema de saúde pública na América Latina. O Benznidazol (BNZ) é utilizado para o tratamento DC e, apesar do seu amplo uso, poucas informações estão disponíveis sobre sua toxicidade e mecanismos das Reações Adversas ao Medicamento (RAM).

**Objetivos:**

Identificar e classificar as reações adversas clínicas e laboratoriais ocasionadas pelo uso do BNZ quanto à sua causalidade e gravidade.

**Métodos:**

Estudo de coorte prospectivo realizado no período de janeiro de 2018 a dezembro de 2021. O acompanhamento do tratamento incluiu consultas e análises laboratoriais antes, 30 e 60 dias após o início do tratamento. As RAM foram classificadas quanto à causalidade e gravidade. Na análise estatística o nível de significância adotado foi p<0,05.

**Resultados:**

Participaram do estudo 40 pacientes com DC crônica, observou-se alta prevalência de RAM com um total de 161 em 30 (90%) pacientes. Destas, 104 (64,6%) foram classificadas como possíveis e 57 (35,4%) como prováveis. As reações foram classificadas em moderadas e leves. Dos 40 pacientes, nove (22,5%) suspenderam o tratamento. As RAM associadas à interrupção e intervenções foram as que afetaram o sistema dermatológico, sistema nervoso central e periférico ou que culminaram em ageusia. Após 30 dias de tratamento, alterações hematológicas e bioquímicas leves foram observadas como linfopenia.

**Conclusão:**

Apesar do elevado percentual de RAM, muitos pacientes foram capazes de completar o tratamento, o que se atribui ao êxito da estratégia de acompanhamento com intervenções de tratamento sintomático juntamente ao aconselhamento, levando à compreensão da sintomatologia e manutenção do tratamento.

## Graphical abstract

**Figura Central:**
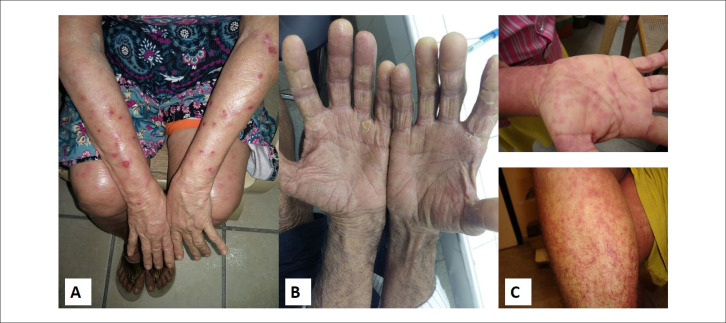
: Causalidade e Gravidade das Reações Adversas e Alterações Laboratoriais ao Tratamento com Benznidazol em Pacientes com Doença de Chagas Crônica Arq Bras Cardiol. 2024; 121(8):e20230787

## Introdução

Para o tratamento etiológico da doença de Chagas (DC), existem dois medicamentos, o Nifurtimox e o Benznidazol (BNZ), sendo este último o único fármaco disponível no Brasil.^1^ O BNZ é disponibilizado pelo Ministério da Saúde apenas para a rede pública e para projetos de pesquisa.^2^

Na fase aguda, o tratamento deve ser realizado em todos os casos imediatamente após a confirmação parasitológica, independentemente da via de transmissão.^3^ Na fase crônica, apesar de sua baixa eficácia, o tratamento também deve ser instituído nos casos recentes (período de cinco a doze anos de infectado), na fase crônica tardia, (acima de doze anos de infectado), na forma indeterminada e nas formas clínicas cardíacas e digestivas leves com objetivo erradicar a infecção, prevenir o aparecimento de lesões nos órgãos ou retardar a evolução das lesões presentes para formas mais graves,^4^ além de interromper a cadeia de transmissão do parasito.^5-8^

O mecanismo de ação do BNZ consiste na formação de radicais livres e/ou metabólitos nucleofílicos a partir da redução do grupo nitro presente na estrutura da molécula do fármaco por ação da nitroredutase.^2^ Esses radicais livres podem lesionar as células do hospedeiro, levando ao surgimento de Reações Adversas a Medicamentos (RAM).^9^

Em 2005, foi criado o centro de referência em assistência farmacêutica às pessoas com DC na Universidade Federal do Ceará, tendo como missão proporcionar ao paciente uma farmacoterapia segura e eficaz, melhorar a qualidade de vida e a adesão ao tratamento, e identificar, prevenir e resolver problemas relacionados a medicamentos. O acompanhamento dos pacientes em tratamento com o BNZ é realizado no Laboratório de Pesquisa em DC – LPDC em parceria com o ambulatório de cardiologia do Hospital Universitário Walter Cantídio (HUWC).

Além de buscar respostas no âmbito da farmacovigilância, esta pesquisa vem mostrar a experiência de um centro, formado por farmacêuticos e médicos cardiologistas, que trata pacientes com DC há mais de 15 anos. Desta forma, o presente estudo buscou avaliar as reações adversas clínicas e laboratoriais ocasionadas pelo uso do BNZ e classificá-las com relação à sua causalidade e gravidade.

## Métodos

Trata-se de um estudo de coorte prospectivo de pacientes com DC crônica em tratamento com BNZ e acompanhados no LPDC da Universidade Federal de Ceará entre janeiro de 2018 e dezembro de 2021.

Foram incluídos no estudo pacientes com idade igual ou superior a 18 anos que chegaram ao LPDC com diagnóstico confirmado para DC (exames sorológicos reagentes por duas metodologias diferentes) e com prescrição de BNZ. Foram excluídos pacientes com tratamento prévio para a DC, gestantes, imunossupressão de qualquer tipo, comorbidades como doenças hepáticas ou renais e pacientes que não compareceram a pelo menos uma consulta farmacêutica durante o tratamento.

### Pacientes e procedimentos

Os pacientes, ao chegarem ao LPDC, foram convidados a participar da pesquisa e, quando de acordo, assinaram um termo de consentimento.

O acompanhamento do tratamento incluiu avaliação clínica (sinais e sintomas) e análises laboratorial (exames hematológicos e bioquímicos) antes do tratamento, e em 30 dias e 60 dias após o início do tratamento. O BNZ usado no estudo foi fabricado pelo Laboratório farmacêutico do Estado de Pernambuco (Lafepe) e fornecido ao LPDC pela Secretaria de Saúde do estado do Ceará.

Como parte do protocolo de acompanhamento farmacêutico, cada paciente foi questionado sobre o aparecimento de RAM a partir do segundo atendimento (em média 30 dias após o início do tratamento) e documentado em formulário específico elaborado pelos farmacêuticos do serviço para notificar os resultados dos exames laboratoriais e a presença de sinais e sintomas clínicos.

Durante todo o tratamento, além do acompanhamento farmacêutico do LPDC, os pacientes receberam acompanhamento médico pelo ambulatório de Cardiologia do HUWC. Além das consultas farmacêuticas e médicas, os pacientes tinham o contato dos pesquisadores para relatar RAM de forma imediata. Ao identificar um paciente com RAM, os profissionais farmacêuticos do LPDC encaminhavam-no ao médico prescritor para as devidas intervenções. Reações adversas específicas foram tratadas clinicamente de acordo com sua gravidade; os eventos leves e moderados foram tratados inicialmente com medicamentos de acordo com os sintomas específicos (analgésicos para cefaleia, inibidores da bomba de prótons para dispepsia, anti-histamínicos e corticoides para alergias). Em alguns casos, o esquema de tratamento foi continuado em paralelo com os medicamentos para os sintomas das reações adversas, e em outros casos a terapia com BNZ foi suspensa temporariamente ou definitivamente. A suspensão temporária do BNZ foi implementada por 10 dias durante o manejo das reações.

Foram avaliados 40 pacientes; desses, 21 (52,5%) apresentavam a forma indeterminada da doença, 14 (35%) a forma cardíaca, quatro (10%) a forma digestiva e um (2,5%) paciente na forma mista, ou seja, apresentava alterações cardíacas e alterações digestivas.

### Adesão ao tratamento e classificação das reações adversas

As reações adversas foram classificadas quanto à causalidade (definida, provável, possível, condicional e não relacionada) e quanto à gravidade (leve, moderada, grave e fatal) e validadas pelo Centro de Farmacovigilância do Ceará (CEFACE) de acordo com metodologia da Organização Mundial de Saúde (WHO). Todas as reações foram classificadas apenas em relação ao BNZ (Tabela 1).

**Tabela 1:** – Classificação das reações adversas quanto à causalidade e quanto à gravidade de acordo com metodologia da Organização Mundial de Saúde (WHO)

Causalidade	Definição
Definida	Um evento clínico relacionado à infusão e/ou reexposição
Provável	Um evento clínico que ocorre onde somente um medicamento pode ser envolvido
Possível	Um evento clínico que ocorre onde dois ou mais medicamentos podem ser envolvidos, ou ainda se pode inferir relação com a doença
Condicional	Um evento clínico em que os dados são parcialmente incompletos ou insuficientes
Não relacionada	Um evento clínico em que não existe uma ligação direta entre a reação adversa e o medicamento, no caso o benznidazol. Assim, essas reações podem estar relacionadas a outros medicamentos, porém são não relacionadas para a causalidade com o benznidazol
**Gravidade**	**Definição**
Leve	Reação de pequena importância clínica e de curta duração, podendo requerer tratamento, não afetando substancialmente a vida do paciente
Moderada	Reação que altera as atividades usuais do paciente, resultando em incapacidade transitória sem sequelas. Necessita de intervenção
Grave	Reação que ameaça diretamente a vida do paciente, provoca hospitalização e pode causar sequelas permanentes
Fatal	Reação que resulta em óbito

*Fonte: World Health Organization.^*7*^*

Um questionário sobre adesão e reações indesejáveis foi aplicado pela equipe do serviço aos 30 e 60 dias. O questionário usado no LPDC é um modelo adaptado por Moreira et al.,^10^ que consiste em perguntas sobre o comportamento do paciente em relação às suas prescrições (número de comprimidos tomados, número de comprimidos não tomados e motivo da não adesão). Além disso, os pacientes foram solicitados a trazer os comprimidos restantes de BNZ para determinar o número de comprimidos que não haviam tomado.

### Acompanhamento laboratorial

Exames bioquímicos para avaliar a função renal (creatinina e ureia) e hepática (alanina aminotransferase - ALT e aspartato aminotransferase - AST) e exames hematológicos (hemograma) foram realizados em todos os participantes antes, durante (30 dias) e após o tratamento (60 dias). Os exames foram realizados pelo Laboratório de Análises Clínicas do HUWC, adotando os valores de referência do fornecedor de cada kit de análise para cada parâmetro analisado.

### Análise estatística

Os dados foram tabulados no Microsoft Office Excel 2016 e analisados utilizando o programa *Graphpad Prism* versão 6.0 e SPSS versão 17. A análise descritiva foi realizada e aplicado teste de normalidade Shapiro-Wilk. O teste exato de Fisher foi aplicado para determinar a associação entre as variáveis categóricas com a ocorrência de reações adversas. As variáveis contínuas foram apresentadas por média ± desvio-padrão e as variáveis categóricas por frequências absolutas e relativas. Para a comparação entre os três momentos da coleta de sangue (antes, 30 e 60 dias de tratamento) aplicou-se a análise da variância unidirecional (*one-way* ANOVA) e o teste de Tukey quando verificado significância a nível de 5% para avaliação dos resultados laboratoriais. Valores de p <0,05 foram considerados estatisticamente significativos.

### Considerações éticas

O estudo foi aprovado pelo comitê de ética em pesquisa que envolve seres humanos do HUWC, sob parecer de número 3.342.170 em 22 de maio de 2019.

## Resultados

### Características sociodemográficas da população estudada

No presente estudo, foram avaliados 40 pacientes, destes 21 (52,5%) eram do sexo masculino com média de idade de 54,6 anos e quanto à escolaridade, 22 (55%) pacientes eram analfabetos/fundamental incompleto. A maioria dos pacientes nasceram (n=32, 80,0%) e continuam morando em cidades do interior do Ceará (n=29, 72,5%), em maior destaque Quixeré, Limoeiro do Norte e Russas.

Alguns pacientes faziam uso de outros medicamentos antes de iniciar o tratamento com BNZ, os quais foram mantidos em concomitância com BNZ, incluindo medicamentos para o tratamento da hipertensão arterial sistêmica, arritmia cardíaca, dislipidemia, diabetes, entre outros.

Durante o tratamento com BNZ, os medicamentos para o sistema cardiovascular foram os mais utilizados (n=45; 47,4%), seguidos de medicamentos para o aparelho digestivo e metabolismo (n=17; 17,9%). Observamos uma média de 2,5 medicamentos por paciente. Nenhum dos medicamentos utilizados apresentava interação medicamentosa conhecida com o BNZ.

### Frequência das reações adversas ao BNZ de acordo com os sistemas ou órgãos

Dos 40 pacientes acompanhados, 30 (75%) apresentaram pelo menos uma reação adversa; 21 finalizaram o tratamento com BNZ e nove suspenderam o tratamento. Dos indivíduos que suspenderam o tratamento, sete suspenderam temporariamente e posteriormente conseguiram concluir a sua terapia, após intervenções farmacêuticas e médicas, e dois suspenderam em definitivo. Durante todo o tratamento, apenas 10 (25%) pacientes não apresentaram RAM.

Nos pacientes que interromperam o tratamento em definitivo, o tempo de tratamento variou de 27 a 40 dias. A dose diária de BNZ foi de 300 mg e todas as RAMdesapareceram após a descontinuação do tratamento.

**Figura 1:**
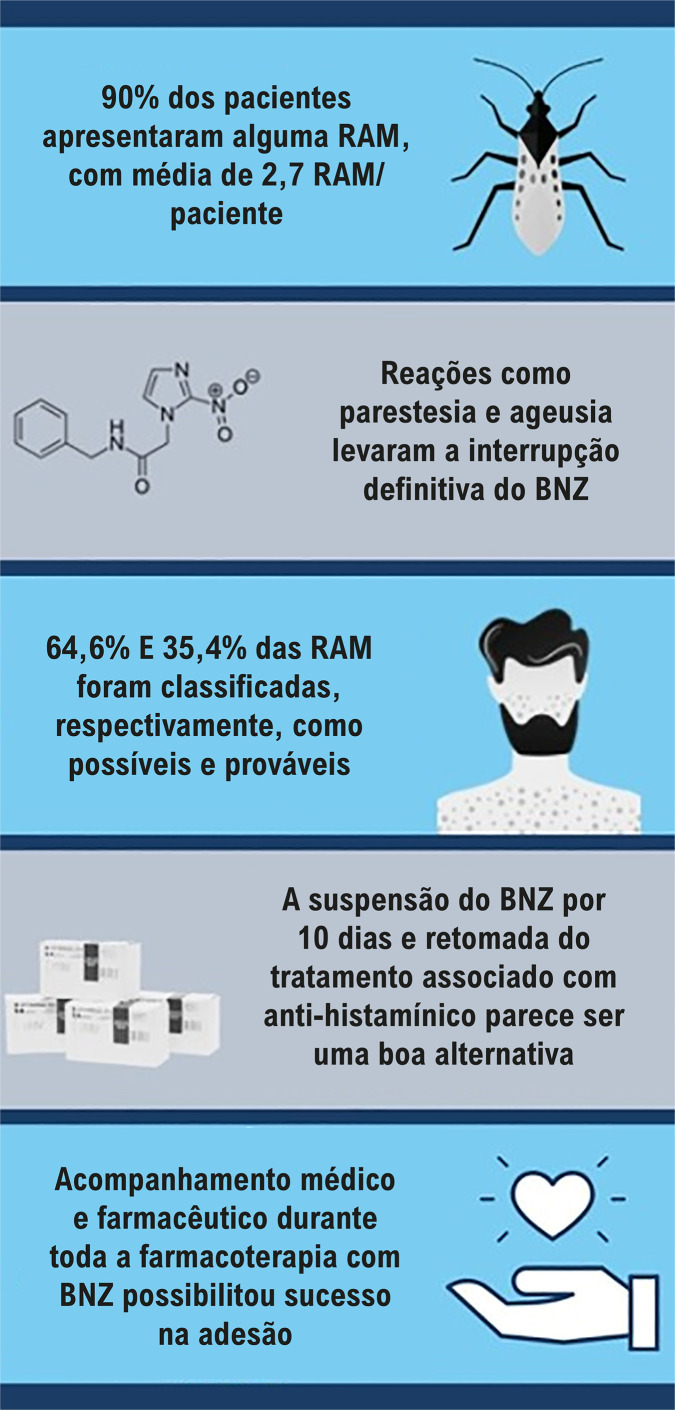
– Reações dermatológicas em pacientes portadores da doença de Chagas crônica tratados com benznidazol e acompanhados pelo Laboratório de Pesquisa em doença de Chagas da UFC. A) Erupção cutânea; B) Descamação das mãos; C) Manchas vermelhas na mão e perna.

As reações adversas mais frequentes foram às relacionadas ao sistema dermatológico como pruridos, descamação da pele e manchas vermelhas/erupções cutâneas (Figura 1); gastrointestinais como dor abdominal, náuseas/enjoo e aumento do apetite. Outro sistema afetado pelo uso do BNZ foi o sistema nervoso central e periférico correspondendo a 11,2% (n=18) das reações adversas encontradas (Tabela 2), destacando-se a parestesia.

**Tabela 2:** – Frequência das Reações Adversas a Medicamentos (RAM) relacionadas ao benznidazol de 40 pacientes com doença de Chagas crônica acompanhados no Laboratório de Pesquisa em Doença de Chagas (LPDC) em Fortaleza entre janeiro/2018 e dezembro/2021

Sistema ou órgão	Frequência absoluta de RAM	% de RAM	Sintomas/Alteração	Frequência de pacientes*	
				n	%
Dermatológico	42	26,1	Manchas vermelhas/ erupções cutâneas	8	20,0
			Manchas roxas	2	5,0
			Prurido	17	42,5
			Pele seca	2	5,0
			Erupções bolhosas	2	5,0
			Hiperemia nas extremidades	1	2,5
			Ardência e queimação na pele	1	2,5
			Descamação da pele	9	22,5
Gastrointestinal	37	23,0	Azia	3	7,5
			Náuseas/enjoos	7	17,5
			Aumento do Apetite	7	17,5
			Dor abdominal	11	27,5
			Dor abdominal + diarreia	4	10,0
			Diarreia	2	5,0
			Vômitos	3	7,5
Estado Geral	23	14,3	Cefaleia	13	32,5
			Algia (dor) nos membros	6	15,0
			Febre	4	10,0
Musculoesquelético	16	9,9	Astenia	11	27,5
			Artralgia	5	12,5
Nervoso central e periférico	18	11,2	Parestesia	10	25,0
			Tontura/Vertigem	8	20,0
Nervoso Autônomo	10	6,2	Perda de apetite	10	25,0
Cardiovascular	8	5,0	Edema nas extremidades/facial	8	20
Órgãos dos sentidos	7	4,3	Ageusia	7	17,5
Total	161	100	Total	161	

*Um mesmo paciente pode apresentar mais de uma reação adversa a medicamentos

Dos nove pacientes que tiveram o tratamento suspenso por 10 dias, sete deles fizeram uso de corticoide (prednisona ou prednisolona) por cinco dias e anti-histamínicos para a resolução da reação adversa cutânea. A ageusia (perda do paladar) foi observada em sete (17,5%) pacientes (Tabela 2), porém, apenas três pacientes relataram essa RAM na primeira etapa do tratamento, o restante relatou a reação após conclusão do mesmo. Em seis pacientes, a falta de paladar foi revertida após a interrupção definitiva ou término do BNZ.

As principais reações responsáveis pela suspensão temporária do BNZ foram as relacionadas ao sistema dermatológico e ao sistema cardiovascular como edema de membros superiores e/ou inferiores. As reações responsáveis pela interrupção definitiva do tratamento foram relacionadas ao sistema nervoso central e periférico como a parestesia e relacionada aos órgãos do sentido como a ageusia.

Deve-se destacar que os indivíduos não apresentavam RAM isoladas ou seja, um mesmo paciente apresentava RAM associadas a vários sistemas.

Com relação ao desenvolvimento de RAM ao BNZ, parece existir distinção entre homens e mulheres, embora não tenha mostrado significância estatística, mas as mulheres mostraram uma tendência maior em desenvolver RAM durante o tratamento em comparação com os homens. Com relação à idade, verificou-se que não existe uma relação significativa entre a idade do paciente e o aparecimento de RAM.

### Causalidade e gravidade das reações adversas

Neste estudo foram identificadas 161 RAM, sendo 104 (64,6%) classificadas como possíveis e 57 (35,4%) como prováveis de terem sido ocasionadas pelo BNZ (Tabela 3). Não foram observadas RAM do tipo definida, condicional e não relacionada (Figura central).

**Tabela 3:** – Classificação das 161 reações adversas ao benznidazol por causalidade e gravidade em 40 pacientes com doença de Chagas que realizaram o tratamento no Laboratório de Pesquisa em Doença de Chagas (LPDC) entre janeiro de 2018 e dezembro de 2021

	Frequência	
Perfil da reação		
**Causalidade**	**n**	**%**
Provável	57	35,4
Possível	104	64,6
Definida	0	0
Condicional	0	0
Não relacionada	0	0
Total	161	100
**Intensidade**	**n**	**%**
Leve	74	46,0%
Moderada	87	54,0%
Grave	0	0
Fatal	0	0
Total	161	100

*Fonte: Próprio autor.*

Além disso, 54% (n=87) das RAM foram classificadas em moderadas (Tabela 3). Pacientes com RAM leve ou moderada tiveram o BNZ suspenso por 10 dias para dar início ao tratamento sintomático e depois foram orientados a reiniciar o tratamento com BNZ.

Pacientes que tiveram o tratamento suspenso devido a reações dermatológicas (12%; n=5), foram submetidos ao tratamento sintomático com corticoide e anti-histamínico e após 10 dias retomaram o tratamento com BNZ associado com anti-histamínico até o final do tratamento. Dessa forma, 60% (n=3) dos que suspenderam o medicamento devido reações dermatológicas conseguiram concluir o tratamento.

As RAM que exigiram a interrupção definitiva do tratamento com BNZ foram classificados em leves como prurido e vômito; moderadas como manchas vermelhas/erupções cutâneas, descamação da pele, parestesia, ageusia e edema nas extremidades/facial. Foi observada uma média de 2,7 RAM por paciente.

### Alterações laboratoriais devido ao uso do Benznidazol

O tratamento da DC com BNZ demanda monitoramento clínico e laboratorial das reações adversas. Antes de iniciar o tratamento, foram realizados exames laboratoriais (hemograma, avaliação de enzimas hepáticas e testes de função renal). Essa conduta foi repetida com 30 e 60 dias do início do tratamento.^11-14^ Na Tabela 4 são observados parâmetros laboratoriais representados por média ± desvio padrão e seu nível de significância.

**Tabela 4:** – Parâmetros hematológicos e bioquímicos dos 40 pacientes com DC tratados com BNZ antes do tratamento, 30 dias e 60 dias após o início do tratamento - LPDC/Fortaleza- janeiro/2018 a dezembro/2021

	Antes do TTO	30 dias de TTO	60 dias de TTO	Valor de referência	Valor p
	Média±DP	Média±DP	Média±DP	Homem/Mulher	
HC (mi/mm^3^)	4,89±0,62	4,83±0,59	4,78±0,61	4,5 a 6,5/ 4,0 a 5,5	0,744^ns^
HB (g/dL)	14,19±1,55	13,91±1,48	14,03±2,08	13,5 a 18 / 12 a 16	0,780^ns^
LEU GLOBAL (/mm^3^)	6323±1619	6388±2116	6335±1750	4.000 a 11.000	0,987 ^ns^
NEU (/mm^3^)	3386±223	3771±279	3542±211	1.600 a 7.500	0,518 ^ns^
BAST (%)	0,10±0,44	0±0	0,24±1,01	0 a 5	0,256^ns^
SEG (%)	52,79±8,79b	58,13±9,68a	55,64±9,15b	40 a 75	0,040*
EO (%)	3,06±1,77	3,58±2,52	3,66±3,07	1 - 6%	0,520^ns^
BASO (%)	0,75±0,43	0,87±9,53	0,63±0,32	0 a 1	0,125^ns^
LINF (%)	35,28+7,73a	28,73±7,53b	31,53±9,25a	20 a 40	0,002**
MONO (%)	8,02±2,07	8,68±2,19	8,26±2,14	2 a 10	0,389^ns^
PLAQ (/mm^3^)	246.325±56055	242.895±65459	238.622±76111	150.000 a 500.000	0,896^ns^
UREIA (mg/dL)	33,28±9,59	32,47±9,78	30,58±10,70	10 a 50	0,493^ns^
CREATININA (mg/dL)	0,91±0,23	0,90±0,19	0,84±0,20	0,7 a 1,3 / 0,6 a 1,1	0,426^ns^
AST (U/L)	24,38±6,52	25,82±9,08	35,81±56,22	<38 / <32	0,712^ns^
ALT (U/L)	27,35±14,95	31,36±22,62	42,58±85,05	< 41 / <31	0,899^ns^

Fonte: Próprio autor. DP: desvio padrão; TTO: tratamento; HC: hemácias; HB: hemoglobina; LEU: leucócitos; NEU: neutrófilos; BAST: bastões; SEG: segmentados; EO: eosinófilos; BASO: basófilos; LINF: linfócitos; MONO: monócitos; PLAQ: plaquetas; ALT: alanina aminotransferase; AST: aspartato aminotransferase; Nível de significância: * p< 0,05; de significância; **p< de 0,01 de significância; ns: não significativo. Colunas com letras iguais não diferem estatisticamente para teste post hoc Tukey, em relação aos dias de tratamento.

Os achados laboratoriais durante o seguimento farmacoterapêutico mostraram casos de alterações hematológicas e alterações bioquímicas leves. Usando o teste ANOVA, detectou-se linfopenia após 30 dias de tratamento com o BNZ (Tabela 4).

As enzimas hepáticas estavam elevadas na seguinte ordem de frequência e variação: AST (n=8; 20%; 39 – 355 U/L) e ALT (n= 10; 25%; 31 – 529 U/L). Foi observado elevação das enzimas hepáticas no 30º dia em sete (53,8%) dos pacientes que apresentaram alteração. Mas essa elevação não foi considerada estatisticamente significativa.

Um dos pacientes que culminou com a suspensão definitiva do BNZ chamou a atenção pelo elevado número de RAM clínicas e laboratoriais como enzimas hepáticas elevadas, alteração no leucograma caracterizada por eosinofilia confirmada em lâmina; RAM relacionada ao estado geral como algia nos membros, reações dermatológicas como prurido e manchas vermelhas, RAM relacionada ao sistema nervoso como parestesia e relacionada aos órgãos do sentido como a ageusia. Todas essas alterações se normalizaram com a suspensão do fármaco e com o tratamento das reações em baixas doses de corticoide por um curto período de tempo, anti-histamínicos, analgésicos e anti-inflamatórios.

Neste estudo, apenas quatro (10%) pacientes apresentaram eosinofilia durante o período de tratamento com o BNZ. É uma alteração laboratorial comum de ocorrer diante de uma reação alérgica, mas não ocorreu em todos os pacientes que apresentaram RAM associadas ao sistema dermatológico.

## Discussão

Os dados encontrados nesse trabalho mostraram uma alta incidência de RAM provocadas pelo uso do BNZ, medicamento de escolha para o tratamento da DC no Brasil e no mundo. Foram abordados aspectos clínicos e laboratoriais dessas reações. Buscou-se esclarecer dúvidas que ainda cercam condutas médicas a respeito da segurança do uso desse fármaco.

Muitos pacientes faziam uso de medicamentos para doenças cardiovasculares, tais como hipertensão arterial sistêmica, insuficiência cardíaca e distúrbios no ritmo cardíaco, e para doenças do trato gastrointestinal e metabolismo como hiperglicemia/diabetes e hipercolesterolemia. Essas comorbidades podem estar relacionadas ao processo de envelhecimento dos pacientes, visto que 14 (35%) tinham idade maior que 60 anos ou atribuídas à fisiopatologia da tripanossomíase.^11,12^ É provável que hipertensão arterial manifeste-se com o avançar da idade independentemente da evolução da DC, porém, mais estudos são necessários para esclarecer melhor essa associação.^12^

No presente estudo, o perfil de reações por intolerância ao BNZ foi semelhante ao relatado em outros estudos observacionais no tratamento da DC. Silva et al.^13^ encontraram uma incidência de RAM de 56,1%, inferior a encontrada em nosso estudo e as RAM que exigiram a interrupção permanente do BNZ foram as dermatoses, (7,1%); distúrbios gastrointestinais (0,7%); e distúrbios do sistema nervoso 28 (1,3%).^14^

Gontijo et al.^15^ observaram em seu estudo uma prevalência de RAM relacionada ao BNZ de 66,1% (n=41), sendo as reações dermatológicas predominantes em 18 (29%) dos 23 (37,1%) pacientes que descontinuaram o tratamento. Os pacientes também fizeram uso de corticoides e anti-histamínicos para a resolução das reações dermatológicas. Adesão do tratamento foi considerada baixa (n=39; 62,9%), quando comparada ao nosso estudo. Diante desses resultados, parece que os corticoides seriam uma opção para o manejo de reações dermatológicas ao BNZ; no entanto, poucos relatos de experiência estão disponíveis sobre a eficácia dos esteroides para prevenir ou conter reações cutâneas devido ao BNZ.^16^

Segundo Salvador et al.,^17^ a reação cutânea associada a BNZ é uma reação adversa medicamentosa compatível com uma reação de hipersensibilidade mediada por células T com uma resposta Th2 produzindo IL-5, com aumento de IL-10. A IL-10 desempenha um papel de modulação da síntese de IL-12 e INF- γ, evitando uma excessiva resposta imune que poderia causar inflamação extensiva e dano aos tecidos do hospedeiro, além de diminuir o desenvolvimento de respostas imunes mediadas por Th2.

Das reações adversas ocasionadas pelo BNZ no sistema nervoso central e periférico destaca-se a parestesia, reação que pode ser irreversível. Esta reação costuma aparecer depois dos 30 dias de tratamento e pode permanecer alguns meses mesmo após a suspensão do BNZ, por isso, a urgência na identificação para iniciar o protocolo de suspensão do BNZ e no tratamento sintomático com medicamentos anticonvulsivantes.^18^ A parestesia apresenta forte impacto sobre a qualidade de vida do paciente. Por isso, a importância de orientar o paciente sobre o possível aparecimento de sensação de dormência ou formigamento em alguma parte do corpo, principalmente nos membros superiores (mãos e braços) ou inferiores (pernas e pés). No presente estudo, os pacientes foram avaliados 30 e 120 dias após o tratamento e os casos de parestesia foram reversíveis.

A ageusia (perda do paladar) é uma reação adversa rara ao BNZ, mas quando presente a recomendação é suspender imediatamente o BNZ.^19^ No nosso estudo, sete pacientes apresentaram ageusia, três pacientes apresentaram essa reação na primeira etapa do tratamento, em que foi possível realizar a intervenção da suspensão imediata do BNZ. Os quatros restantes relataram a reação após conclusão do tratamento. A perda do paladar foi reversível em seis pacientes após a interrupção definitiva ou término do tratamento. Os órgãos dos sentidos como olfação e gustação são vitais, sua perda traz graves consequências na qualidade de vida, bem como pode representar risco à saúde do indivíduo.^20^ Os medicamentos costumam afetar mais a gustação que a olfação.^19,20^ O paladar pode ser afetado em casos de lesões do nervo facial proximal à saída da corda timpânica.^21^

Muitos estudos relatam que as mulheres apresentam mais reações adversas ao BNZ que os homens. Essa diferença deve ser atribuída a fatores anatômicos e fisiológicos, presentes nas mulheres, que podem afetar o processo farmacocinético dos fármacos.^8,22,23^

No presente estudo foi encontrado 161 RAM, sendo 54% (n=87) classificadas em moderadas, podendo resultar em incapacidade transitória sem sequelas e necessitando de intervenção (administração de medicamentos e suspensão do tratamento). Olivera et al.^22^ apresentou resultados semelhantes quanto à gravidade das RAM, sendo 57,4% leves e 35,5% moderadas; já a causalidade as reações foram diferentes do nosso estudo apresentando maior quantidade de reações definidas, mas muitas reações foram classificadas em prováveis e possíveis.^22^ Gontijo et al.^15^ também encontraram alta prevalência de reações prováveis (80,9%).

Silva et al.^13^ classificaram as RAM em seu estudo como leves (n=900; 43,4%) e moderadas (n=263; 12,7%), diferindo dos nossos achados. Não foi observado RAM grave e fatal no seu estudo, o que concorda com nossos achados. Apesar do alto índice de RAM relacionadas ao BNZ, houve predominância de reações consideradas moderadas (54%; n=87) e leves (46%; n=74) em nosso estudo; dessa forma, podemos considerar que o BNZ é um medicamento seguro para o tratamento da DC, salvo os casos em que o BNZ não é indicado.^19,24^

A suspensão do BNZ por 10 dias e a retomada do tratamento associado com anti-histamínicos parece ser uma boa alternativa para a conclusão do tratamento da DC na presença de reações adversas dermatológicas.

Diante da alta frequência reações adversas, o sucesso da adesão ao BNZ no presente estudo deve-se à importância do acompanhamento médico e farmacêutico durante todo o tratamento. O acompanhamento farmacêutico do tratamento com BNZ realizado pelo LPDC/UFC pode prevenir ou detectar precocemente as reações adversas antes de se tornarem graves.^25,26^ Isso pode justificar o sucesso da adesão (n=38; 95%) ao tratamento neste estudo quando comparado às taxas de adesão nos estudos de Gontijo et al.^15^ (62,9%), Olivera et al.^22^ (76,8%) e Hasslocher-Moreno et al.^8^ (68,9%), e também a não observação de reações adversas graves ou fatais.

Durante o tratamento com BNZ é necessário monitoramento laboratorial para verificar a função hepática (AST e ALT) e renal (ureia e creatinina) dos pacientes.^27^ A biotransformação do fármaco ocorre no tecido hepático, com posterior eliminação pelos rins.^28^ Quando um aumento plasmático dessas enzimas atinge 10 ou mais vezes os valores de referência, pode ser reflexo de hepatite causada por drogas. No presente estudo, os valores atingiram um máximo de 9,3 U/L para AST, 12,9 U/L para ALT. Pavan et al.^27^ observaram valores máximos de 9,1 U/L para ALT, 8,2 U/L para AST, resultados muito semelhantes ao encontrado no presente estudo. Também encontram em seu estudo um paciente com uma alta concentração de enzimas hepáticas, se destacando dos demais. Este paciente também apresentou sintomas digestivos, reação cutânea e alteração hematológica caracterizada por neutropenia. Após suspensão do BNZ e uso de corticoides as alterações desapareceram.

Estudos experimentais em animais realizado por Garcia et al.^29^demonstraram que não há necrose produzida pelo BNZ. Isso pode justificar o fato de as enzimas hepáticas voltarem aos valores normais após o término ou suspensão do tratamento. Ainda não se conhece claramente os mecanismos envolvidos nas alterações hepáticas induzidas pelo BNZ, mas a elevação tardia das enzimas sugere que deve haver uma relação com o aumento do tempo de exposição ao fármaco.

A presença de eosinófilos na pele é comum em distúrbios dermatológicos associados a RAM e tem sido levantada a hipótese de que os eosinófilos podem contribuir para a defesa contra o patógeno e regular as respostas inflamatórias.^30,31^Nas reações a medicamentos, os eosinófilos intervêm nas urticárias, angioedema, e na síndrome de DRESS – *Drug Reaction* (*or Rash*) *with Eosinophilia and Systemic Symptoms –* caracterizada por reação sistêmica medicamentosa que pode ser muito grave. A imunomodulação das reações alérgicas com eosinofilia faz com que os corticoides e anti-histamínicos aumentem a apoptose dos eosinófilos.^32^ Isso justifica a melhora e resolução das reações adversas apresentadas pelos pacientes do presente estudo ao utilizarem o esquema prednisona 20 mg por dia durante cinco dias e anti-histamínicos como loratadina 10 mg por dia ou dicloridrato de hidroxizina 25 mg duas a três vezes ao dia por 10 dias ou em concomitância com o uso do BNZ.

Dos pacientes que suspenderam o tratamento com BNZ devido à RAM, sete conseguiram retomar e concluir o tratamento usando anti-histamínicos em concomitância e apenas dois tiveram o tratamento interrompido definitivo. Rodríguez-Guardado et al.^33^ observaram que a combinação de doses crescentes de BNZ e anti-histamínico oral (dexclorfeniramina) foi capaz de evitar distúrbios da pele e permitir um curso completo de 60 dias de tratamento com BNZ em 19 pacientes.

A reação idiossincrática ao BNZ parece ser o mecanismo mais provável responsável pelas reações adversas, uma vez que há mais de um órgão/sistema do organismo afetado, além disso, observa-se a recuperação rápida das reações adversas após a retirada da droga.^17^ Não há estudos clínicos na literatura que consigam responder diretamente os mecanismos que envolvem as reações adversas induzidas por BNZ,^27^ por isso mais estudos devem ser realizados para esclarecer os mecanismos envolvidos para traçar estratégias no tratamento e acompanhamento dos pacientes que necessitarem utilizar esse medicamento, uma vez que não temos outras opções disponíveis no mercado farmacêutico.

As limitações do estudo foram o número limitado de consultas de seguimento (apenas duas, sendo a primeira, 30 dias após o início do tratamento) e o reduzido número de pacientes devido à suspensão dos atendimentos farmacêuticos no LPDC/UFC causada pela pandemia da Covid-19 por mais de um ano.

## Conclusão

O trabalho demonstrou uma alta incidência de RAM, sendo a maioria de gravidade moderada, com o uso do BNZ no tratamento da DC (Figura Central).

As reações mais frequentes foram dermatológicas, gastrointestinais e do sistema nervoso. As RAM mais associadas à interrupção e intervenções foram descamação da pele, prurido, erupções bolhosas, a parestesia e ageusia. As alterações laboratoriais da função hepática, induzidas pelo BNZ, foram leves e facilmente controláveis, devendo ser monitoradas principalmente no 30º dia e ao final do tratamento. Não ocorreram óbitos e não houve necessidade de internação durante a terapia com BNZ.

Apesar do elevado percentual de RAM, muitos pacientes foram capazes de completar o tratamento, o que se atribui ao êxito da estratégia de acompanhamento com intervenções de tratamento sintomático juntamente ao aconselhamento, levando à compreensão da sintomatologia e manutenção do tratamento.
