# Supplementary material for: Causalidade e Gravidade das Reações Adversas e Alterações Laboratoriais ao Tratamento com Benznidazol em Pacientes com Doença de Chagas Crônica
Source: Arq Bras Cardiol. 2024 Sep 6;121(8):e20230787. [Article in Portuguese] doi: 10.36660/abc.20230787 (PMC11495806; doi:10.36660/abc.20230787)
Supplement: Translation

# Translation: Causality and Severity of Adverse Reactions and Biochemical Changes to Benznidazole Treatment in Patients with Chronic Chagas Disease

**DOI:** 10.36660/abc.20230787i

**Authors:** Alanna Carla da Costa Belmino, Emanuela Kelly Silva Sousa, José Damião da Silva, Eduardo Arrais Rocha, Francisca Mylena Melgaço Nunes, Tiago Lima Sampaio, Leones Fernandes Evangelista, Bruna Ribeiro Duque, Isabel Cristina da Silva Araújo, José Igor de Oliveira Jacó, Maria de Fátima Oliveira

**Affiliations:** 1 Brazil Universidade Federal do Ceará, Fortaleza, CE – Brazil

**Keywords:** Chagas Disease, Drug-Related Side Effects and Adverse Reactions, Laboratory Examinations and Diagnostics

## Abstract

**Background:**

Chagas disease (CD) is a serious public health problem in Latin America. Benznidazole (BNZ) is used for the treatment of CD and, despite its wide use, little information is available about its toxicity and mechanisms of adverse drug reactions (ADR).

**Objectives:**

To identify and classify clinical and laboratory adverse reactions caused by BNZ in terms of causality and severity.

**Methods:**

Prospective cohort study from January 2018 to December 2021. Treatment follow-up included visits and biochemical tests (complete blood count, liver and kidney function tests) before, during and after treatment. ADR were classified according to causality and severity. In the statistical analysis, the significance level was set at p<0.05.

**Results:**

Forty patients with chronic CD were included. A high prevalence of ADR was observed 161 ADR in 30 patients [90%]; of these, 104 (64.6%) were classified as possible and 57 (35.4%) as probable. The ADR were classified as moderate and mild. Of the 40 patients, nine (22.5%) discontinued treatment. ADR associated with treatment discontinuation and interventions were those that affected the dermatological system, central and peripheral nervous system and sense organs such as ageusia. Mild hematological and biochemical changes such as lymphopenia were observed after 30 days of treatment.

**Conclusion:**

Many patients were able to complete the treatment even with ADR, which can be attributed to the successful follow-up strategy with symptomatic treatment and counseling, leading to patient’s awareness of symptoms and treatment adherence.

## Introduction

Two drugs can be used for the etiological treatment of Chagas disease (CD) – Nifurtimox and Benznidazole (BNZ) – and the latter is the only one available in Brazil.^1^ The Brazilian Ministry of Health approves the use of BNZ in the public health and in research projects.^2^

In the acute phase of the disease, treatment should be started immediately after parasitological confirmation, regardless of the transmission route.^3^ In the chronic phase, despite its low efficacy, treatment should be implemented in more recent cases (five-12 years of infection), in late chronic phase (> 12 years of infection), in the indeterminate form and in mild digestive and cardiac forms of the disease. The treatment aims at eradicating the infection, preventing target-organ damage or the progression of existing lesions to more severe conditions,^4^ and disrupting the parasite transmission chain.^5-8^

The mechanism of action of BNZ consists of the formation of free radicals and/or nucleophilic metabolites from the reduction of a nitro group in the drug molecule by the action of nitroreductase enzymes.^2^ These free radicals may damage the host cells, and trigger adverse drug reactions (ADR).^9^

In 2005, a referral center for CD patients was created at the Federal University of Ceara. The aim of this center was to provide CD patients with safe and effective pharmaceutical care, improve quality of life and treatment adherence, and to identify, prevent and solve drug-related problems. Patient treatment with BNZ and follow-up is conducted at the Research Laboratory for CD (RLCD) of the Federal University of Ceara in partnership with Walter Cantidio University Hospital.

This study not only sought answers to pharmacosurveillance questions but also presented results of a center, composed of pharmacists and cardiologists, with over 15 years’ experience in the treatment of CD. Thus, the present study aimed to assess clinical and laboratory adverse reactions to BNZ and classify them by cause and severity.

## Methods

This was a prospective cohort study of patients with chronic CD treated with BNZ and seen at the at the RLCD of the Federal University of Ceara between January 2018 and December 2021.

Patients aged 18 years or older, referred to the RLCD with a confirmed diagnosis of CD (by two serological methods) and prescribed BNZ. Patients previously treated for CD, pregnant women, immunosuppressed patients, those with comorbidities (e.g. liver or kidney disease) and patients who missed at least one pharmaceutical visit during treatment were excluded.

### Patients and procedures

After arriving at the laboratory facilities, patients were invited to participate in the study, and those who agreed to participate signed an informed consent form.

During follow-up, patients were assessed for clinical signs and symptoms, and underwent laboratory tests (hematological and biochemical) before treatment, and at 30 days and 60 days. BNZ was manufactured at the Pernambuco State Pharmaceutical Laboratory and provided to the RLCD by the Secretary of Health of Ceara.

As part of the pharmacotherapeutic follow-up protocol, each patient was asked about ADR from the second visit on (mean of 30 days after treatment onset), which was registered in a specific form, developed by the pharmacists of the center, to document the results of laboratory tests and the presence of signs and symptoms.

During the treatment, in addition to pharmacotherapeutic follow-up at RLCD, the patients were also seen at the cardiology outpatient center of Walter Candidio University Hospital. In addition, all participants could contact the researchers to report eventual ADR immediately. When a patient presenting ADR was identified by the LPDC pharmacists, the patient was referred to the preceptor who decided about the most appropriate interventions. Specific adverse reactions were treated medically according to their severity; mild and moderate events were initially treated by symptom – analgesics for headache, proton pump inhibitors for dyspepsia, and antihistamines and corticoids for allergies). In some cases, the treatment scheme was continued in parallel with the drugs used for the adverse reaction symptoms and, in others, BNZ therapy was discontinued, either temporarily or permanently. Temporary suspension of BNZ was implemented for 10 days during the management of reactions.

Forty patients were included; 21 (52.5%) of them had the indeterminate form of CD, 14 (35%) the cardiac form, four (10%) the digestive form, and one the mixed form, *i.e*., had both cardiac and digestive abnormalities.

### Treatment adherence and classification of adverse reactions

Adverse reactions were classified according to causality (certain, probable, possible, conditional and unrelated) and validated by the Ceara Pharmacosurveillance Center (CEFACE) based on the methods recommended by the World Health Organization. All reactions were classified in relation to BNZ only (Table 1).

**Table 1:** – Classification of adverse reactions by causality and severity according to the World Health Organization

Causality	Definition
Certain	A clinical event related to drug infusion and/or re-exposure
Probable	Clinical event related to only one drug
Possible	Clinical event that may be related to two or more dugs; or when a relationship with the disease may be inferred
Conditional	Clinical event with partially incomplete or insufficient data
Unrelated	Clinical event with no direct relationship between the adverse reaction and the drug (in this case, benznidazole). Thus, these reactions may be related to other drugs, but not to benznidazole for causality
**Severity**	**Definition**
Mild	Event of little clinical relevance and short duration, may require treatment but does not affect patient’s life substantially
Moderate	Event that affects patient’s usual activities, resulting in transient disability without sequelae; requires intervention
Severe	Life-threatening reaction that leads to hospitalization and may cause permanent sequelae
Fatal	Reaction that results in death

*Source: World Health Organization.^*7*^*

A questionnaire on drug adherence and undesirable reactions was administered by the service staff at 30 and 60 days. This questionnaire, used at the RLCD, was adapted by Moreira et al.,^10^ and consists of questions about patient behavior regarding drug prescriptions (number of pills taken, number of pills not taken, and reasons for non-adherence). Besides, all patients were instructed to bring the remaining BNZ pills to determine the number of pills not taken.

### Laboratory follow-up

Biochemical tests to assess renal (creatinine and urea) and liver (alanine aminotransferase, ALT, and aspartate aminotransferase, AST) functions, and hematological tests (total blood count) were performed before, during (30 days) and after (60 days) treatment. All tests were carried out by the clinical analysis laboratory, and the reference values provided by the assay kit manufacturer were adopted.

### Statistical analysis

Data were organized in a Microsoft Office Excel spreadsheet and analyzed using the *Graphpad Prism* version 6.0 and SPSS version 17. Descriptive analysis was conducted, and the Shapiro-Wilk test applied. The Fisher’s exact test was used to determine the association between categorical variables and the occurrence of adverse events. Continuous variables were described as mean and standard deviation and categorical variables as absolute and relative frequency. For comparison between the blood collection times (before, 30 and 60 days after treatment), the one-way ANOVA was used. When statistical differences (p<0.05) were found for laboratory data, the Tukey test was also used. A p<0.05 was set as statistically significant.

### Ethical aspects

The study was approved by the ethics committee of Walter Cantidio University Hospital (approval number 3342170, 22 May, 2019).

## Results

### Sociodemographic characteristics of the study population

Forty patients were evaluated; 21 (52.5%) were male, mean age was 54.6 years. Regarding educational attainment, 22 (55%) were illiterate or had some elementary school. Most patients were born (n=32, 80.0%) and still live in the state of Ceara (n=29, 72.5%), especially Quixere, Limoeiro do Norte and Russas.

Some patients had been using other medications before starting BNZ treatment; these drugs were not discontinued and used concomitantly with BNZ, including medications for hypertension, arrhythmias, dyslipidemias, diabetes, among others.

During treatment with BNZ, the most frequently used drugs were medications for cardiovascular disease (n=45; 47.4%), followed by gastrointestinal and metabolic diseases (n=17; 17.9%). There was a mean of 2.5 medications per patient. None of the medications used had known drug interactions with BNZ.

### Frequency of adverse reactions to BNZ by organ or system

Of the 40 patients included, 30 (75%) had at least one adverse reaction. Twenty-one completed BNZ treatment and nine discontinued the treatment. Of these patients who discontinued treatment, seven discontinued treatment temporarily and were able to conclude it later after pharmacotherapeutic and medical interventions, and two patients discontinued treatment permanently. During treatment, only 10 (25%) patients did not have ARDs.

Among patients who discontinued treatment permanently, the overall treatment time varied from 27 to 40 days. Daily dose of BNZ was 300 mg, and all ARDs disappeared after treatment discontinuation.

The most common adverse reactions were those related to the skin, including pruritus, skin desquamation, dermatological system and skin rashes/eruptions (Figure 1), gastrointestinal reactions including abdominal pain, nausea, and increased appetite. In addition, the central and peripheral nervous systems were affected by BNZ, accounting for 11.2% (n=18) of the adverse reactions (Table 2), including paresthesia.

**Figure 1:** Dermatological reactions in patients with chronic Chagas disease treated with benznidazole and monitored by the UFC Chagas Disease Research Laboratory. A) Skin rash; B) Peeling of the hands; C) Red spots on the hands and legs.

**Table 2:** – Frequency of benznidazole-induced adverse drug reactions (ADR) in 40 patients attending the research laboratory for Chagas disease in Fortaleza between January 2018 and December 2021

System or organ	Absolute frequency of ADR	ADR (%)	Symptoms/Alterations	Patients (%)*	
				n	%
Dermatological	42	26.1	Skin rashes / eruptions	8	20.0
			Purple spots	2	5.0
			Pruritus	17	42.5
			Dry skin	2	5.0
			Blisters	2	5.0
			Hyperemia of extremities	1	2.5
			Burning sensation	1	2.5
			Skin desquamation	9	22.5
Gastrointestinal	37	23.0	Heartburn	3	7.5
			Nausea	7	17.5
			Increased appetite	7	17.5
			Abdominal pain	11	27.5
			Abdominal pain + diarrhea	4	10.0
			Diarrhea	2	5.0
			Vomiting	3	7.5
General	23	14.3	Headache	13	32.5
			Limb pain	6	15.0
			Fever	4	10.0
Musculoskeletal	16	9.9	Asthenia	11	27.5
			Arthralgia	5	12.5
Central and peripheral nervous system	18	11.2	Paresthesia	10	25.0
			Dizziness/vertigo	8	20.0
Autonomic nervous system	10	6.2	Loss of appetite	10	25.0
Cardiovascular	8	5.0	Edema of extremities /face	8	20
Sense organs	7	4.3	Ageusia	7	17.5
Total	161	100	Total	161	

*One patient may present more than one adverse drug reaction

Of the nine patients who discontinued treatment for 10 days, seven used corticosteroids (prednisone or prednisolone) for five days and antihistamines for cutaneous adverse drug reactions. Ageusia (total loss of taste) was observed in seven (17.5%) patients (Table 2), however, only three reported this adverse reaction in the beginning of treatment. The others reported ageusia after treatment conclusion. In six patients, loss of taste was reversed after permanent or transient discontinuation of BNZ.

The main ADR responsible for the temporary suspension of BNZ were dermatological reactions and cardiovascular reactions like upper and lower limb edema. The reactions responsible for permanent treatment discontinuation were those related to the central and peripheral nervous system, like paresthesia and those related to organ senses like ageusia.

It is worth mentioning that the patients did not have adverse reactions alone, *i.e*., the same patient had ADR associated with various systems.

With respect to the development of ADR to BNZ, there may be a difference between men and women, although not statistically significant, but women showed a greater tendency to develop ADR during treatment as compared with men. Regarding age, there was no significant relationship between age and the occurrence of ADR.

### Cause and severity of adverse reactions

In this study, 161 ADR were identified; 104 (64.6%) were classified as possibly ADR and 57 (35.4%) as probably caused by BNZ (Table 3). No certain, conditional or unrelated ADR was found (Central Illustration).

**Table 3:** – Classification of the 161 adverse reactions to benznidazole in 40 patients attending the research laboratory for Chagas disease in Fortaleza between January 2018 and December 2021, by causality and severity

	Frequency	
Adverse reaction classification		
**Causality**	**n**	**%**
Probable	57	35.4
Possible	104	64.6
Certain	0	0
Conditional	0	0
Unrelated	0	0
Total	161	100
**Intensity**	**n**	**%**
Mild	74	46.0
Moderate	87	54.0
Severe	0	0
Fatal	0	0
Total	161	100

*Source: Authors.*

In addition, 54% (n=87) of ADR were classified as moderate reactions (Table 3). Patients with mild or moderate ADR discontinued BNZ for 10 days to initiate symptom treatment and were then instructed to resume treatment.

Patients who discontinued treatment due to skin reactions (n=5; 12%) underwent symptom treatment with corticosteroids and antihistamines, and 10 days later resumed treatment with BNZ combined with antihistamines until the end of treatment. Thus, 60% (n=3) of patients who discontinued treatment due to skin reactions were able to complete the treatment.

The ADR that led to permanent discontinuation of BNZ were classified as mild such as pruritus and vomiting; moderate, such as skin rashes/eruptions, skin desquamation, paresthesia, ageusia, and edema of the distal extremities and face. A mean of 2.7 ADR per patient were observed.

### Biochemical changes caused by BNZ

The use of BNZ in the treatment of CD requires clinical and laboratory monitoring of adverse reactions. Before starting treatment, laboratory tests (total blood count, hepatic enzyme activity, and renal function test) were performed. This was repeated at 30 days and at 60 days of treatment.^11-14^ Laboratory parameters, expressed as mean and standard deviation, and the level of significance, are described in Table 4.

**Table 4:** – Hematological and biochemical parameters of 40 patients treated with benznidazole at three timepoints: before, at 30 days and 60 days of treatment; laboratory for Chagas disease, Fortaleza, Brazil, January 2018 - December 2021

	Before	At 30 days	At 60 days	Reference value	p Value
	Mean ± SD	Mean ± SD	Mean ± SD	Men/women	
RBC (mi/mm^3^)	4.89±0.62	4.83±0.59	4.78±0.61	4.5 – 6.5/ 4.0 – 5.5	0.744^ns^
HB (g/dL)	14.19±1.55	13.91±1.48	14.03±2.08	13.5 - 18 / 12 - 16	0.780^ns^
WBCs (/mm^3^)	6323±1619	6388±2116	6335±1750	4,000 - 11,000	0.987 ^ns^
NEU (/mm^3^)	3386±223	3771±279	3542±211	1,600 – 7,500	0.518 ^ns^
BAND (%)	0.10±0.44	0±0	0.24±1.01	0 - 5	0.256^ns^
SEG (%)	52.79±8.79b	58.13±9.68a	55.64±9.15b	40 - 75	0.040*
EO (%)	3.06±1.77	3.58±2.52	3.66±3.07	1 - 6%	0.520^ns^
BASO (%)	0.75±0.43	0.87±9.53	0.63±0.32	0 - 1	0.125^ns^
LYMPHO (%)	35.28+7.73a	28.73±7.53b	31.53±9.25a	20 - 40	0.002**
MONO (%)	8.02±2.07	8.68±2.19	8.26±2.14	2 - 10	0.389^ns^
PLATELETS (/mm^3^)	246.325±56055	242.895±65459	238.622±76111	150,000 – 500,000	0.896^ns^
UREIA (mg/dL)	33.28±9.59	32.47±9.78	30.58±10.70	10 - 50	0.493^ns^
CREATININA (mg/dL)	0.91±0.23	0.90±0.19	0.84±0.20	0.7 - 1.3 / 0.6 - 1.1	0.426^ns^
AST (U/L)	24.38±6.52	25.82±9.08	35.81±56.22	<38 / <32	0.712^ns^
ALT (U/L)	27.35±14.95	31.36±22.62	42.58±85.05	< 41 / <31	0.899^ns^

SD: standard deviation; RBC: red blood cells; Hemoglobin; WBCs: white blood cells; NEU: Neutrophiles; SEG: Segmented; EO: Eosinophiles; BASO: Basophiles; LYMPHO: Lymphocytes; MONO: Monocytes; ALT: alanine aminotransferase; AST: aspartate aminotransferase; Level of significance: *p< 0.05; **p< 0.01; ns: non-significant. Same letter indicates no significant differences by post-hoc Tukey test.

During follow-up, the laboratory findings revealed mild hematological and biochemical alterations. The ANOVA showed occurrence of lymphopenia after 30 days of BNZ (Table 4).

Elevations in hepatic enzymes were observed, in order of frequency and variation: AST (n=8; 20%, 39 – 355 U/L) and ALT (n= 10; 25%; 31 – 529 U/L). Elevations in hepatic enzymes were found on the 30^th^ day in seven (53.8%) of the patients who showed these alterations, although these elevations were not statistically significant.

One patient, who discontinued BNZ permanently, attracted attention for the high number of clinical and biochemical ADR, including elevated hepatic enzymes, altered white blood cell count (eosinophilia confirmed by microscopy); ADR related to general health like limb pain, skin reactions like pruritus and rashes, ADR related to the nervous system like paresthesia and other reactions of the sense organs like ageusia. All these changes were normalized with BNZ discontinuation and use of low-dose corticosteroid for a short period, in addition to antihistamines, analgesics and anti-inflammatories.

In this study, only four patients (10%) showed eosinophilia during treatment with BNZ. It frequently occurs in allergic reactions, although it was not detected in all patients who experienced ADR in the skin.

## Discussion

Results of the present study showed a high incidence of adverse reactions to BNZ, which is the drug of choice for CD in Brazil and in the world. Clinical and biochemical aspects of these reactions were approached. We also sought answers that still affect medical decisions regarding the safety of this new drug.

Many patients used medications for cardiovascular diseases, such as systemic hypertension, heart failure and heart rhythm disorders, and for gastrointestinal and metabolic disease, such as hyperglycemia/diabetes and hypercholesterolemia. These comorbidities may be either related to aging of these patients, since 14 (35%) were older than 60 years, or attributed to the pathophysiology of trypanosomiasis.^11,12^ It is probable that, with aging, patients develop hypertension regardless of the course of CD; however, more studies are needed to elucidate such association.^12^

The profile of adverse reactions to BNZ described in the present study was similar to that reported in other observational studies on the management of CD. Silva et al.^13^ found an incidence of 56.1% of ADR, lower than the incidence found in our study, and the ADR that led to permanent discontinuation of BNZ were dermatosis (7.1%), gastrointestinal disorders (0.7%), and nervous system disorders.^14^

Gotijo et al.^15^ reported a prevalence of BNZ-related ADR of 66.1% (n=41), and skin reactions were predominant in 18 (29%) of the 23 (37.1%) patients that discontinued treatment. The patients also used corticosteroids and antihistamines to treat these reactions. Treatment adherence was considered low (n=39, 62.9%) as compared to our study. Based on these findings, corticosteroids may be used in the management of skin reactions to BNZ. However, there are few reports available on the efficacy of steroids in preventing or reducing skin adverse reactions to BNZ.^16^

According to Salvador et al.,^17^ cutaneous reactions associated with the use of BNZ are ADR compatible with a T-cell mediated hypersensitivity reaction with a Th2 response, producing IL-5, with an increase in IL-10. IL-10 plays a role in modulating the synthesis of IL-12 and INF-γ, preventing an excessive immune response that could cause severe inflammation and damage of host tissue, and the development of Th2-mediated immune response.

Paresthesia is among the adverse reactions induced by BNZ in central and peripheral nervous system. It usually appears after 30 days of treatment, may persist for months after BNZ discontinuation and can even be irreversible. Therefore, urgency is needed to identify the symptom and initiate the BNZ discontinuation protocol and treatment with anticonvulsant agents.^18^ Paresthesia has a strong impact on patient quality of life. For this reason, patients should be warned of the possibility of numbness or tingling sensation in any part of the body, especially in upper (hands and arms) or lower (legs and feet) limbs. In the present study, patients were evaluated 30 and 120 days after treatment and the cases of paresthesia were reversible.

Ageusia (complete loss of taste) is a rare adverse reaction to BNZ, but, when present, BNZ should be immediately suspended.^19^ In our study, seven patients had ageusia, three of them during the first phase of treatment, when the immediate discontinuation of the BNZ could be made. The other four patients reported ageusia after treatment completion. The symptom was resolved after permanent discontinuation of treatment or its completion. Organs of senses like smell and taste are vital; losing any of them may have severe consequences in the quality of life and represents a life-threatening condition.^20^ The taste is usually more severely affected by these medications than the smell;^19^ it can also be altered by damage in the anterior segment of the chorda tympani.^21^

Several studies have reported that adverse reactions to BNZ are more common in women than men. This may be explained by anatomical and physiological factors, present in women, that may affect the whole pharmacokinetic process of the drugs.^8,22,23^

In the present study, 161 ADR were found, 54% (n=87) classified as moderate, potentially leading to a transient disability, with no sequelae, but requiring some type of intervention (administration or discontinuation of drug treatment). Oliveira et al.^22^ showed similar results regarding the severity of ADR – 57.4% mild ADR and 35.5% moderate ADR – however, the causes of ADR were different, with more cases of certain adverse reactions and many cases of probable and possible reactions.^22^ Gotijo et al.^15^ also reported a high prevalence of probable reactions (80.9%).

In the study by Silva et al.,^13^ the ADR were classified as mild (n=900; 43.4%) and moderate (n=263; 12.7%), which was different from our findings. They did not find any severe or fatal ADR, in accordance with our study. Despite the high occurrence rate of adverse reactions to BNZ in our study, they were predominantly moderate (n=87; 54%) and mild (n=74; 46%) reactions; therefore, BNZ may be considered a safe drug to use in the treatment of CD, except in case of contraindication of BNZ.^19,24^

BNZ discontinuation for 10 days and resume of treatment combined with antihistamines seems to be a good strategy to complete the treatment of CD in the presence of adverse skin reactions.

Considering the high frequency of adverse reactions, the successful treatment adherence in this study can be attributed to the medical and pharmacotherapeutic follow-during the whole treatment with BNZ. The pharmacotherapeutic follow-up during BNZ treatment carried out at the RLCD/UFC may prevent or early detect the adverse reactions before they become severe.^25,26^ This can explain the success of BNZ therapy adherence in our study (n= 38; 95%) as compared with the adherence rates reported by Gontijo et al.^15^ (62,9%), Oliveira et al.^22^ (76,8%) and Hasslocher-Moreno et al.^8^ (68,9%), and also the non-observation of severe or fatal reactions.

During therapy with BNZ, biochemical monitoring is essential, to assess hepatic function (AST and ALT) and renal function (urea and creatinine) of patients.^27^ Biotransformation of the drug occurs in the hepatic tissue followed by renal excretion.^28^ A plasma increase of these enzymes by at least 10-fold the reference values may be a reflection of drug-induced hepatitis. In the present study, the highest values for AST and ALT were 9.3 U/L and 12.9 U/l, respectively. Pavan et al.^27^ observed maximum ALT and AST values of 9.1 U/L and 8.2 U/L, respectively, which are similar to our results. They also found a patient with high concentrations of hepatic enzymes, distinguishing from the others. This patient also had digestive symptoms, cutaneous reaction and hematological alterations characterized as neutropenia. After BNZ discontinuation and use of corticosteroids, these alterations disappeared.

Animal studies conducted by Garcia et al.^29^ demonstrated that there is no case of necrosis induced by BNZ. This may explain the normalization of hepatic enzyme levels after treatment completion or discontinuation. The mechanisms involved in hepatic alterations induced by BNZ have not been fully elucidated, but the later increase in enzyme levels suggests a relationship with the time of drug exposure.

The presence of eosinophils on the skin is common in dermatological disorders associated with ADR, and the hypothesis has been raised that these cells contribute to host defense against the pathogen and regulate inflammatory responses.^30,31^ In ADR, eosinophils act by stimulating pruritus, angioedema, and the DRESS – Drug Reaction (or rash) with Eosinophilia and Systemic Symptoms – characterized by a potentially severe systemic drug reaction. Because of the immunomodulation of the allergic reactions with eosinophilia, corticosteroids and antihistamines increase eosinophil apoptosis.^32^ This explains the improvement and resolution of adverse reactions presented by the patients in the current study after using BNZ combined with prednisone 20 mg for five days and antihistamines like loratadine 10 mg per day or hydroxyzine 25 mg twice or three times a day for 10 days.

Among the patients who discontinued treatment with BNZ due to ADR, seven resumed and completed treatment using antihistamines and only two had their discontinued treatment permanently. Rodríguez-Guardado et al.^33^observed that the combination of increasing doses of BNZ with oral antihistamine (dexchlorpheniramine) could prevent skin disturbances and contributed to completion of the 60-day treatment with BNZ by 19 patients.

An idiosyncratic reaction to BNZ seems to be the most likely mechanism responsible for the adverse reactions, since more than one organ or system is involved, and the adverse reactions are rapidly resolved after drug withdrawal.^17^ There are no clinical studies in the literature describing the mechanisms involved in the adverse reactions induced by BNZ.^27^ For this reason, further studies are needed to elucidate these mechanisms so as to develop strategies in the treatment and in the follow-up of patients who need this drug, which is the only drug commercially available.

Limitations of this study included the reduced number of follow-up visits (only two visits, the first being 30 days after treatment onset) and the small number of patients due to disruption in health services at the RLCC/UFC for more than one year caused by the COVID-19 pandemic.

## Conclusion

This study showed a high incidence of ADR, mostly of moderate severity, with the use of BNZ in CD treatment (Central Illustration). The most common reactions were cutaneous, gastrointestinal and nervous system reactions. The main ADR that led to treatment discontinuation and interventions were skin desquamation, pruritus, blisters, paresthesia and ageusia. Changes in liver function biochemical tests induced by BNZ were mild and easily controlled, and should be monitored on the 30^th^ day and at the end of treatment.

Despite the high frequency of ADR, several patients completed therapy, which may be attributed to the successful follow-up strategy with interventions for symptom management and counseling, resulting in patient awareness of symptoms and treatment adherence
